# A novel graphene-based micro/nano architecture with high strength and conductivity inspired by multiple creatures

**DOI:** 10.1038/s41598-021-80972-8

**Published:** 2021-01-14

**Authors:** Muzhi Li, Xiuya Wang, Ru Zhao, Yuanyuan Miao, Zhenbo Liu

**Affiliations:** 1 Key Laboratory of Bio-Based Material Science and Technology of Ministry of Education, Northeast Forestry University, Harbin, 150040 P. R. China; 2 Key Laboratory of Forest Plant Ecology, Ministry of Education, Northeast Forestry University, Harbin, 150040 P. R. China

**Keywords:** Electronic properties and devices, Mechanical and structural properties and devices, Electronic properties and devices, Mechanical and structural properties and devices

## Abstract

In the long history of development and elimination, the creatures have derived a variety of exquisite structures and unique properties, typically natural nacre, marine mussel and Glycera to adapt to the environment and resist the predation of the enemy. Hence, inspired by the combination of special structures and properties of multiple creatures, a novel type of graphene-based micro/nano architecture was proposed, and the related bioinspired nanocomposites were fabricated, Polydopamine coated Graphene oxide/Nanocellulose/Polydopamine (P-GCP). Apart from replicating the layered structure of natural nacre, P-GCP also introduced copper ions and polydopamine to simulate the hardening mechanism of the Glycera’s jaw and the composition of adhesive proteins in mussels to further improve the tensile strength and conductivity of nanocomposites, respectively. The test results showed that the tensile strength of P-GCP reached 712.9 MPa, which was 5.3 times that of natural nacre. The conductivity of artificial nacre was as high as 207.6 S/cm, which was equivalent to that of reduced graphene oxide (rGO). Furthermore, the material exhibited outstanding electrical conductivity when it connected as wires in a circuit, demonstrating the practical application prospects in aerospace, supercapacitors, biomaterials, artificial bones and tissue engineering.

## Introduction

Over billions of years of development and evolution, nature has created and optimized a variety of biological tissues and materials including natural nacre, marine mussel and Glycera. Natural nacre is mainly composed of calcium carbonate sheets (95 vol.%) and also contains small amounts of organic polymers such as β-chitin and silk fibroin^1,2^. It has a characteristic inorganic–organic layered structure, which achieves the unity of mechanical strength and toughness^3,4^. Recently, a variety of artificial nacres were designed and prepared based on different 2D inorganic building blocks such as calcium carbonate (CaCO_3_)^5^, aluminium oxide (Al_2_O_3_)^6,7^, layered double hydroxides (LDH)^8,9^, nanoclay^10^, and double-walled carbon nanotubes (DWNT)^11,12^. Compared to other 2D inorganic platelets, graphene-based materials have excellent tensile strength, electronic transmission performance, and optical transmittance, as well as large specific surface areas^13,14^. Graphene oxide (GO), which inserted various oxygen-containing groups on its surface^15,16^. Duan et al.^17^ prepared reduced graphene oxide/nanofibrillar cellulose/10,12-pentacosadiyn-1-ol (rGO-NFC-PCDO) ternary artificial nacre with GO as building blocks. With a GO content of 95 wt.%, the mechanical strength and toughness of the composites were 314.6 MPa and 9.8 MJ/m^3^, respectively.

The special protein secreted by mussel’s cells is considered to have super strong adhesion properties^18^. Inspired by the composition of adhesive proteins in mussels, dopamine (DA) is frequently used as a binder coating for substrates. DA, one kind of organic micromolecules containing various functional groups, which could spontaneously develop into polydopamine (PDA) on effect of functional groups in molecules under alkalescence condition^19^. In addition to the superior adhesion performance, PDA coating also has abundant catechol groups that could introduce functional molecules to the surface of the material through secondary reaction. The jaws are generally considered to be the hardest part of Glycera, which is mainly related to the formation of a cross-linked molecular network between copper ions and proteins^20^. Glycera's jaw is mainly composed of 43 wt.% protein, 39 wt.% melanin, and a small amount of copper, the latter of which is present in the near-tip regions of the jaw in the form of [Cu_2_(OH)_3_Cl] fibers as reported in the literature^21^. Interestingly, the copper is concentrated at the tip of the jaw instead of being evenly distributed, which enhances the material's hardness, stiffness and abrasion resistance^22,23^.

In this work, a novel micro/nano architecture was proposed inspired by various creatures, and related materials were obtained. Based on the "brick–mortar" structure of natural nacre, P-GCP artificial nacre exhibited hierarchical micro/nanoscale architecture. Cross-linked molecular network and copper nanoparticle coating inspired by Glycera and marine mussels increased the tensile strength and electrical conductivity of the materials, respectively. Furthermore, the synergistic effects of different interface interactions including hydrogen bonding, ionic bonding, covalent bonding and chelate architecture, were also crucial factors in improving mechanical strength^24^. The tensile strength of P-GCP had reached 712.9 MPa, which were 5.3 times that of natural nacre. The successful connection of nanocomposites with conductivity of 207.6 S/cm in the circuit indicated huge application prospects in diverse electronic devices.

## Methods

### Materials

Graphite crystals (99 wt.%) were purchased from Jiangsu Changjia Degaoxin Carbon Material Co., Ltd. Sodium nitrate (NaNO_3_) was purchased from Shanghai Yixin Chemical Co., Ltd. Potassium permanganate powders (KMnO_4_) was purchased from Qufu Xinxin Chemical Co., Ltd. H_2_SO_4_ (98 wt.%), and hydrogen peroxide (H_2_O_2_ 30 wt.%) were purchased from Anshan Anji Chemical Co., Ltd. Deionized water, distilled water and absolute ethanol were purchased from Sigma-Aldrich (China). Cupric chloride dihydrate (CuCl_2_·2H_2_O), sodium phosphate monobasic monohydrate (NaH_2_PO_4_·H_2_O), dopamine hydrochloride, and tris (hydroxymethyl) aminomethane hydrochloride (Tris HCl) were purchased from Shanghai McLean Biochemical Technology Co., Ltd. Potassium hydroxide (KOH) was purchased from Zibo Zesheng Chemical Co., Ltd. Cellulose nanofiber (CNF) was purchased from Zhengzhou Yuyan New Building Material Co., Ltd.

### Preparation of GO

115 mL of concentrated H_2_SO_4_ (98wt.%) was placed into a flask immersed in an ice water bath (0C), and subsequently graphite crystals (5 g) and NaNO_3_ (2 g) was added into H_2_SO_4_ solution with continuous stirring. Immediately after, KMnO_4_ (15 g) was mixed to the above suspension slowly and carefully, which was proceeded for 80 min at 2-10C. The mixture was transferred to an oil bath (30-40C) and the reaction was continued for 40 min. After the procedure was completed, the resultant mixture was removed from oil bath, and then permitted to add deionized water (220 mL) drop by drop under continuous stirring. The temperature of the mixture was controlled at 90-100C, at which time the color changed from brownish yellow to reddish brown. After the suspension was naturally cooled to room temperature, H_2_O_2_ was added until there were no bubbles. Finally, the pure graphene oxide nanosheets were obtained after ultrasound, centrifugation and dialysis.

### Preparation of GO/PDA composites

1.576 g of Tris HCl particles were dissolved in 50 mL of distilled water, and an appropriate amount of KOH solution was added to stabilize the pH at 8.5. The Tris buffer suspension was obtained and stored at room temperature. The prepared Tris buffer suspension and dopamine solution(2 mg/ml) were poured into the uniform GO solution in turn, and the pH of the mixed suspension was fixed. After constant stirring for 36 h, Da self polymerization into PDA and the liquor changed from brownish yellow to dark black. Repeated the sonication and centrifugation procedures to obtain homogeneous suspension. Finally, the suspension was transferred to an oven at 65C, and the GO/PDA composites were obtained after 12 h. The composites, GO/PDA-1, GO/PDA-2, GO/PDA-3, and GO/PDA-4, were prepared in GO: DA mass ratios of 95:5, 90:10, 85:15, and 80:20, respectively.

### Preparation of PDA-coated GO/CNF (P-GC) films

Firstly, the as-prepared CNF dispersion was added to the GO solution, stirred continuously at room temperature until the suspension was homogeneous. Then, the GO/CNF films were prepared by vacuum-assisted filtration. Finally, the obtained films were immersed in the PDA solution with pH = 8.5, and after 36 h, washed and dried to obtain P-GC films. Four kinds of P-GC films were fabricated with different CNF, named as P-GC-1 (GO: CNF = 95:5), P-GC-2 (GO: CNF = 90:10), P-GC-3 (GO:CNF = 85:15) and P-GC-4 (GO:CNF = 75:25).

### Preparation of PDA-coated GO/PDA (P-GP) films

The prepared Tris buffer solution and dopamine solution were added to the uniform GO solution in turn, and the pH of the mixed suspension was fixed. After constant stirring for 36 h, the homogeneous suspension was obtained by repeating the sonication and centrifugation procedures. The GO/PDA films were obtained by vacuum-assisted filtration and drying. Finally, the GO/PDA films were separately immersed in the PDA solution and Cu^2+^ solution respectively for the same time, and washed with absolute ethanol to obtain P-GP. The films, P-GP-1, P-GP-2, P-GP-3, and P-GP-4, were prepared in GO: DA mass ratios of 95:5, 90:10, 85:15, and 75:25, respectively.

### Preparation of P-GCP

The as-prepared CNF dispersion and GO solution were successively added to the Tris buffer suspension, and the pH of the suspension was adjusted to 8.5. Subsequently, DA was added and stirred at room temperature for 36 h. Repeated the sonication and centrifugation procedures to obtain GO/CNF/PDA homogeneous suspension. The GO/CNF/PDA composites were prepared by vacuum-assisted filtration and drying. Finally, they were separately immersed in the PDA solution and Cu^2+^ solution and washed with absolute ethanol to obtain P-GCP. In this work, four kinds of P-GCP ternary artificial nacres with 5 wt.% PDA were prepared: P-GCP-1 (GO:CNF = 95:5), P-GCP-2 (GO:CNF = 90:10), P-GCP-3 (GO:CNF = 85:15), and P-GCP-4 (GO:CNF = 75:25), respectively.

### Characterization

Scanning electron microscopic (SEM) images were obtained using a QUANTA200 instrument. Fourier transform infrared spectroscopy (FT-IR) was performed on a Spectrum 400 instrument over the scan range 550–4000 cm^−1^. Atomic force microscopic (AFM) images were obtained using a Bruker Dimension Icon instrument. X-ray diffraction (XRD) analysis was carried out using a XRD-6100 over the scan range 5–60° and scan speed of 5°/min. All the X-ray photoelectron spectroscopic (XPS) measurements were taken using an ESCALab220i- XL instrument (Thermo Scientific) with a monochromatic Al Kα X-ray source. The mechanical properties were measured in tensile mode using an AI-7000S TC160701511 tester at a loading rate of 1 mm/min with a gauge length of 5 mm. All the samples were cut into strips, 20 mm long and 3 mm wide, before conducting the measurements, and the test results were the average measurements values for all the samples. The thicknesses of all the samples were confirmed using a thickness gauge. The values of tensile strength and strain were derived from the stress–strain curves. The values of toughness were obtained by calculating the integral areas under the curves. A standard two-probe method using a source meter (CHI760E) was employed to measure the electrical conductivities of the artificial nacre specimens.

## Results and Discussion

**Figure 1:**
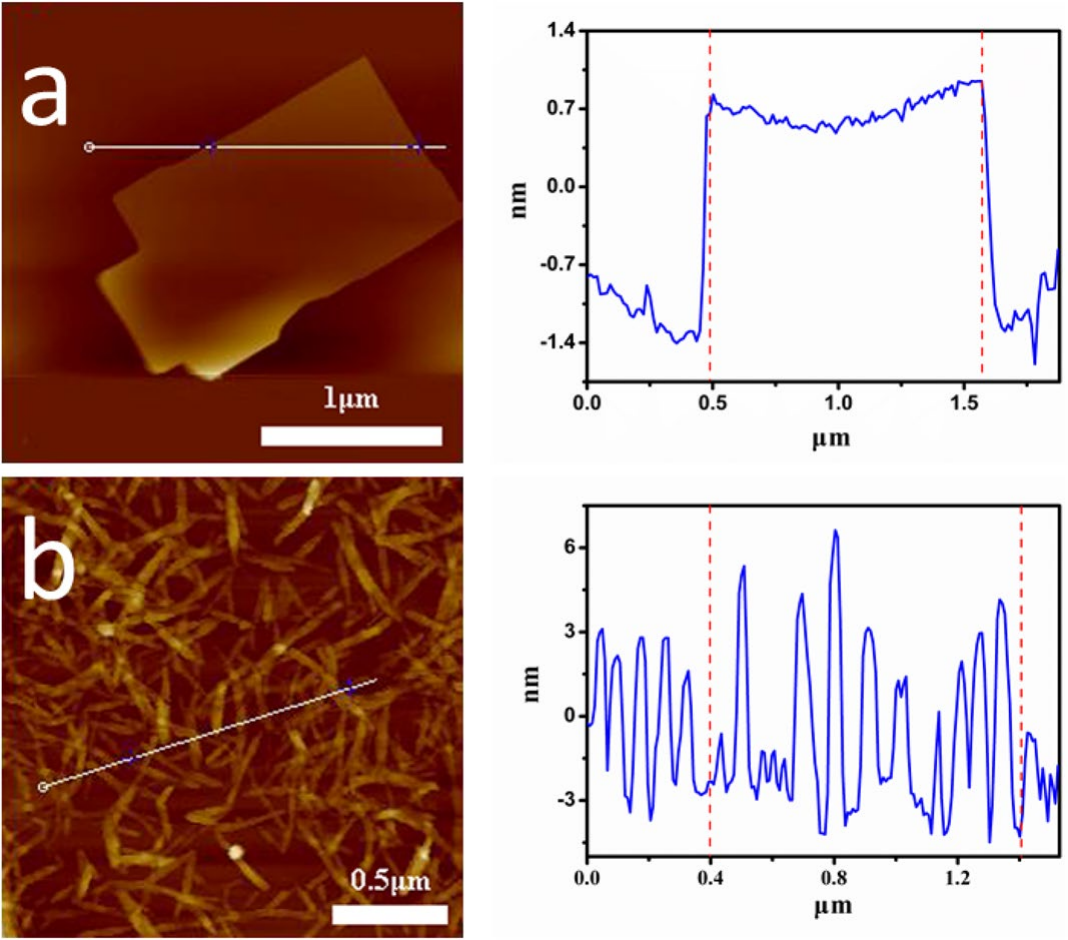
(**a**) Size of GO nanosheets, and (**b**) rod-like nanocellulose.

Figure 1 shows the image of graphene oxide and cellulose nanofibers under the atomic force microscope(AFM). The prepared graphene oxide showed a single-layer structure, and the cellulose nanofibers showed an obvious rod-like structure. The material is sufficient and complete, and then suitable for subsequent preparation and research.

**Figure 2:**
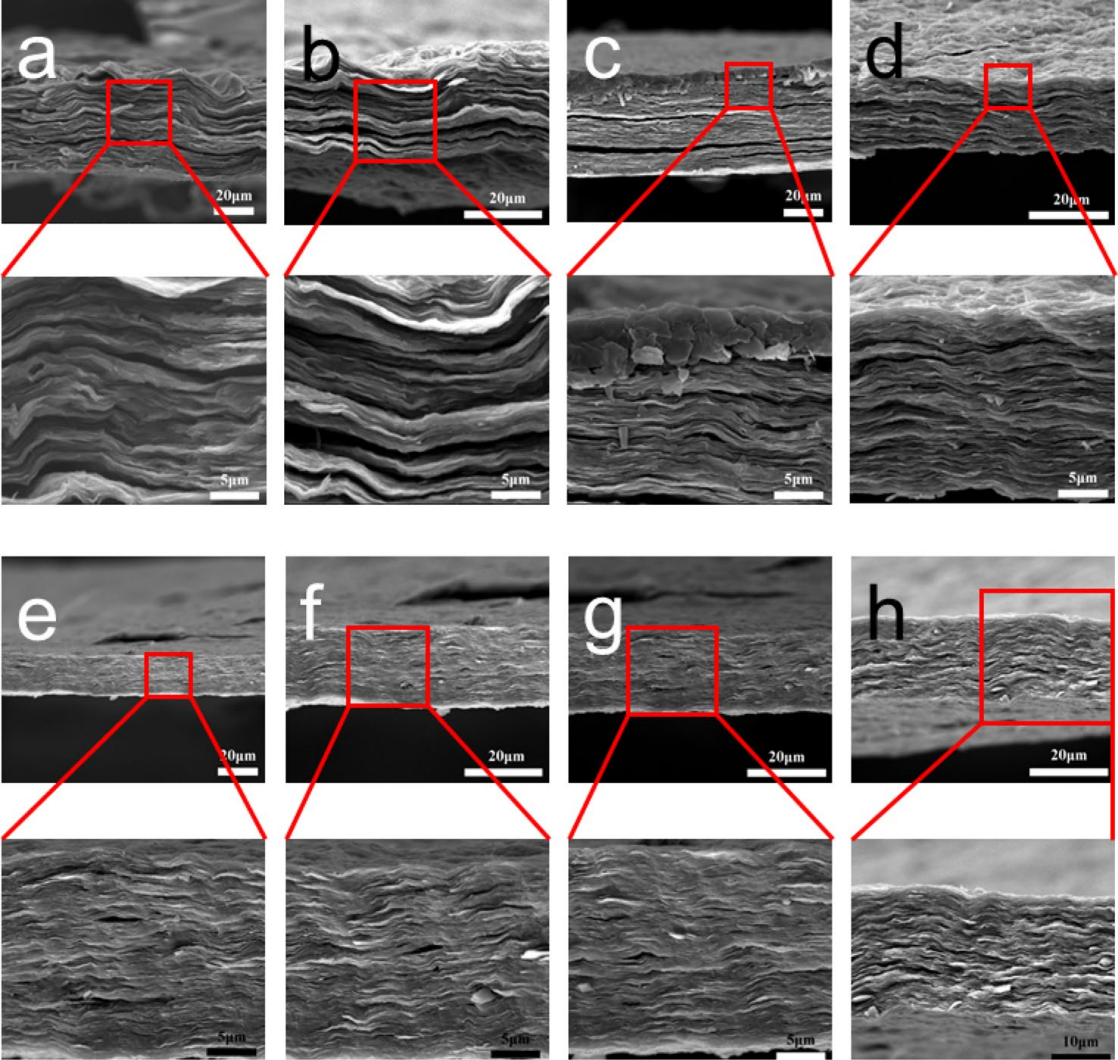
Fracture morphologies of (**a**) P-GP-1, (**b**) P-GP-2, (**c**) P-GP-3, (**d**) P-CP-4, (**e**) P-GCP-1, (**f**) P-GCP-2, (**g**) P-GCP-3, and (**h**) P-GCP-4 artificial nacres.

**Figure 3:**
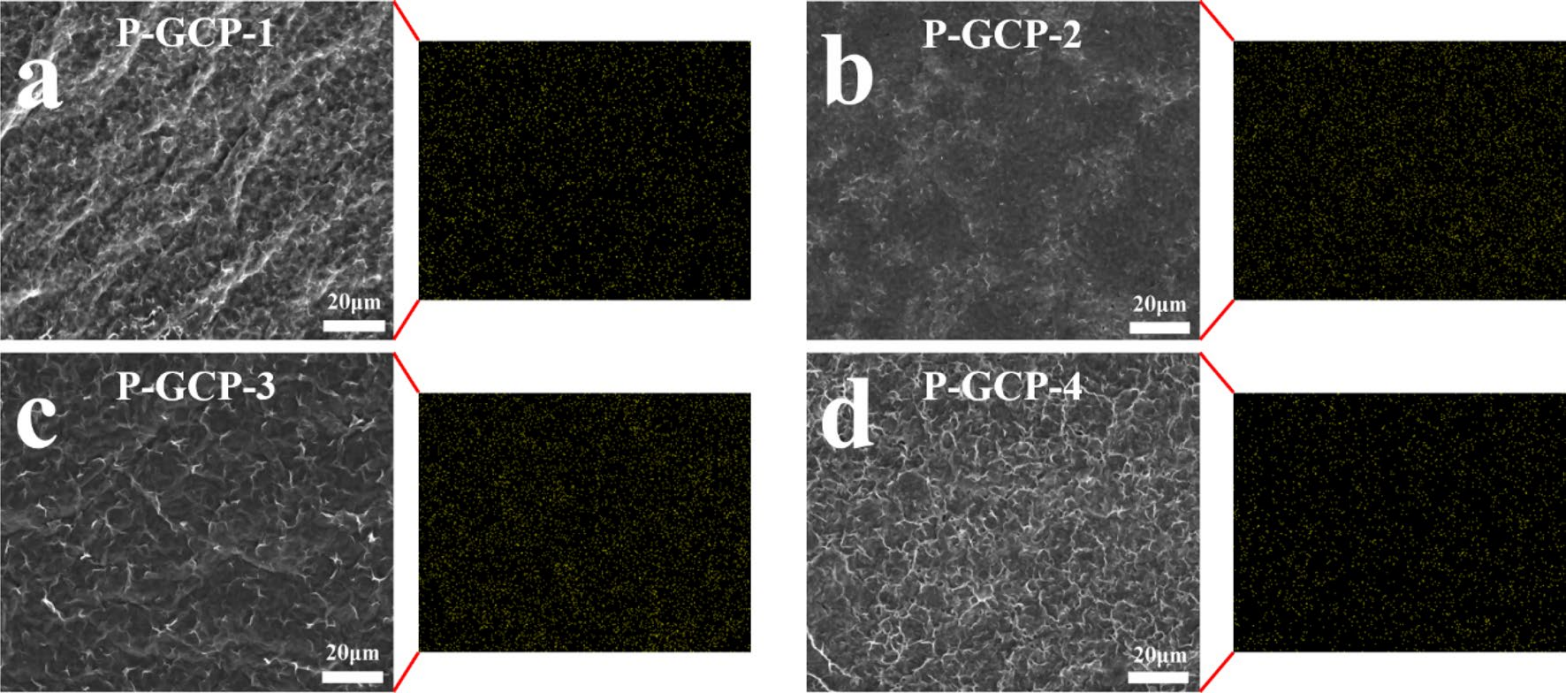
Corresponding EDS of Cu element originating from CuCl_2_ in P-GCP, revealing that copper nanoparticles are homogeneously distributed without aggregation.

Both the fracture morphologies of P-GP and P-GCP artificial nacre showed a characteristic layered structure (Fig. 2). Energy dispersive X-ray spectroscopic (EDS) measurements were performed on its surface, as shown in Fig. 3. The result of EDS analysis found a large amount of elemental copper in the materials, indicating that copper nanoparticles were uniformly dispersed on the nanocomposites^25^.

**Figure 4:**
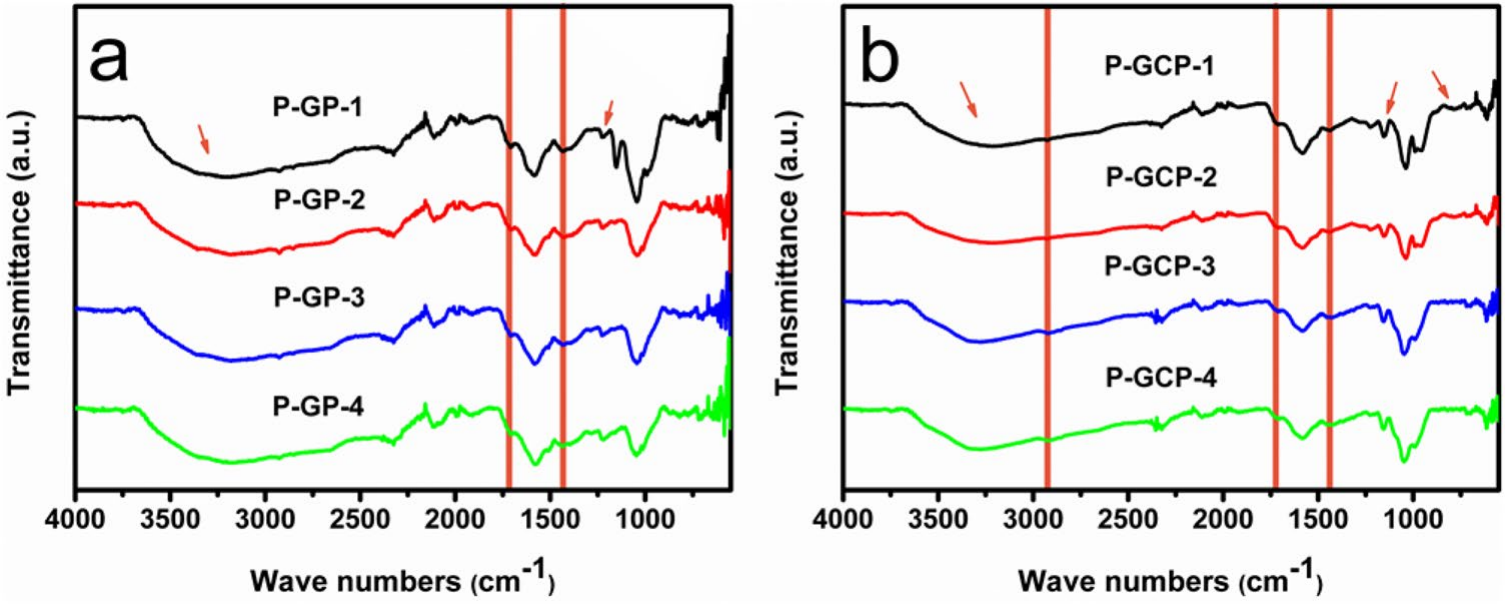
FT-IR spectra of (**a**) P-GP binary artificial nacre and (**b**) P-GCP ternary artificial nacre.

To verify the interactions between GO and PDA and the addition of nanocellulose, a large number of characterizations were implemented. The Fourier transform infrared (FTIR) measurements were performed to identify the cross-linking between GO and PDA, as shown in Fig. 4a. Different from GO/PDA composites, the infrared spectrum of the P-GP artificial nacre changed little with the increase of dopamine addition^26^. In P-GP-1, the stretching vibration peak of the C = O bonds at 1730 cm^−1^ and the stretching peak of the C-O bonds at 1346 cm^−1^ had reached the minimum; the characteristic peaks of the epoxy groups at 870 cm^−1^ and 1220 cm^−1^ disappeared; the absorption band at 3600–2600 cm^−1^ reached the widest, indicating that the surface-coated polydopamine also reacted with graphene oxide to saturate the reaction. In Fig. 4b, the absorption band at 3600–2600 cm^−1^ was continuously deepened due to the hydroxyl groups attached to the nanocellulose. In addition, the characteristic peaks at 1430 cm^−1^, 1375 cm^−1^ and 897 cm^−1^ correspond to the in-plane bending vibration peaks of HCH and OCH bonds, the bending vibration peaks of C-H bonds and the vibration of C_1_, respectively, confirming the addition of nanocellulose.

**Figure 5:**
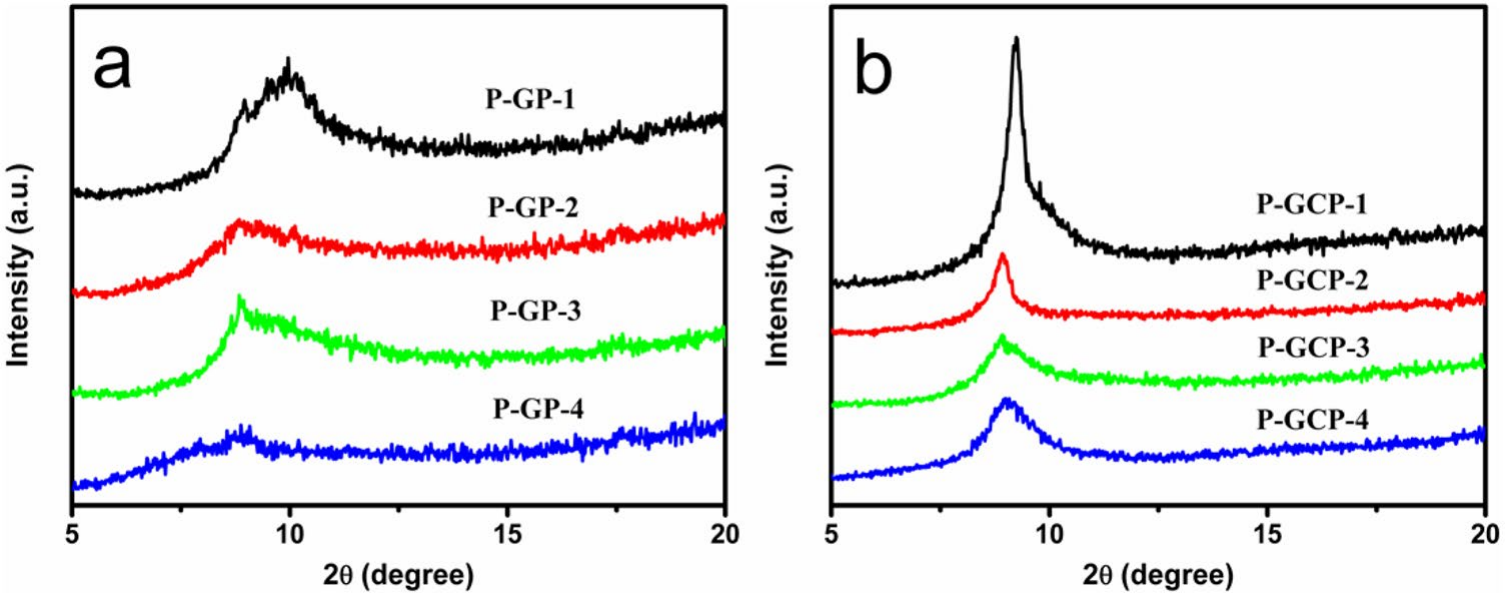
(**a**) XRD patterns of P-GP binary artificial nacre nanocomposites, and (**b**) XRD patterns of P-GCP ternary artificial nacre nanocomposite.

The results of XRD analysis confirmed that DA and CNF were successfully introduced into the interlayers of the GO nanosheets (Fig. 5). The specific values of the d-spacing of P-GP artificial nacreous nanocomposites and P-GCP artificial nacreous nanocomposites have been shown in Table 1. In previous study, the (002) diffraction peak of GO appeared at 11.20°, and the interlayer distance (d-spacing) of the corresponding GO nanosheets was about 7.89 Å, which was similar to that of the reference^27^. In the P-GP artificial nacre (Fig. 5a), when the PDA content was 5 wt.%, the d-spacing of P-GP-1 was about 8.87 Å, which was slightly higher than that of GO, indicating that the PDA had successfully penetrated the GO nanosheets. With increase in PDA content, the intensity of the diffraction peak was slightly reduced. With the addition of nanocellulose, the intensity of diffraction peaks is significantly reduced, the 2θ value shifts to the left, and the interlayer spacing of graphene oxide increases. When the amount of nanocellulose added was 10 wt%, the interlayer spacing reached the maximum, which was 9.90 Å (2θ = 8.92°), indicating that nanocellulose successfully entered between the graphene oxide layers. With the continuous addition of nanocellulose, the intensity of the diffraction peak does not change much, the 2θ value shifts to the right, and the interlayer spacing of graphene oxide decreases. When the amount of nanocellulose added was 25 wt%, the interlamellar spacing dropped to 9.81 Å (2θ = 9.01°), indicating that the content of nanocellulose in the interlamellar layer has reached saturation, and excessive nanocellulose will be deposited on the material Surface, resulting in the continuous reduction of the interlayer spacing^28^. After polydopamine is coated, nanocellulose is hidden under the coating, so the presence of surface nanocellulose cannot be observed on the SEM picture. Even if the lamella spacing is reduced to 9.81 Å, it is still higher than the 9.55 Å (2θ = 9.25°) of P-GCP-1, indicating that the lamella is still mixed with a large amount of nanocellulose.

**Table 1:** The d-spacing of P-GP artificial nacre nanocomposites and P-GCP artificial nacre nanocomposites.

Sample	2*θ* (°)	d-spacing (Å)
P-GP-1	9.96	8.88
P-GP-2	8.78	10.06
P-GP-3	8.84	9.99
P-GP-4	8.88	9.94
P-GCP-1	9.25	9.55
P-GCP-2	8.92	9.90
P-GCP-3	8.93	9.89
P-GCP-4	9.01	9.81

**Figure 6:**
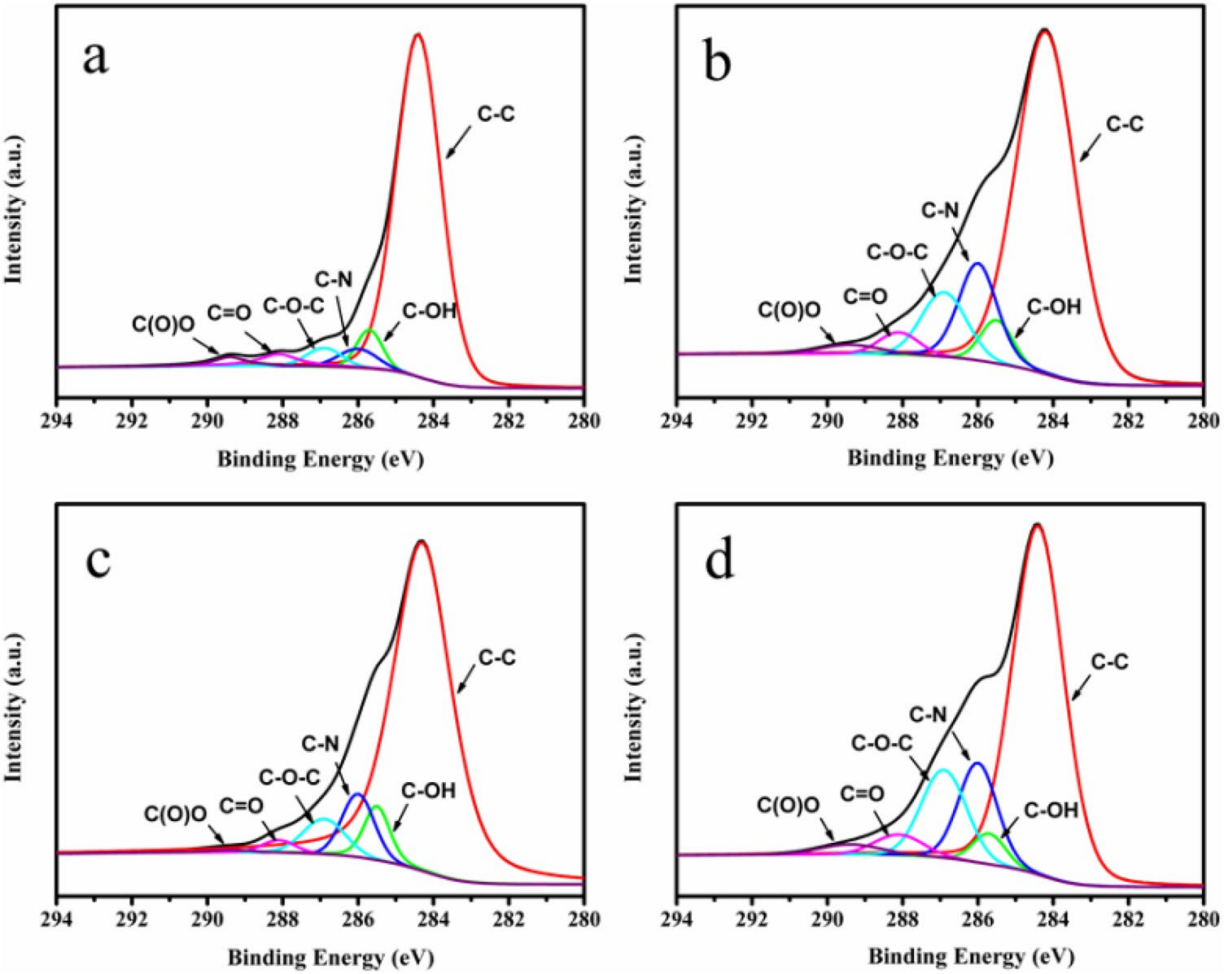
XPS spectra of (**a**) P-GP-1, (**b**) P-GP-2, (**c**) P-GP-3, and (**d**) P-GP-4 binary artificial nacre nanocomposites.

**Figure 7:**
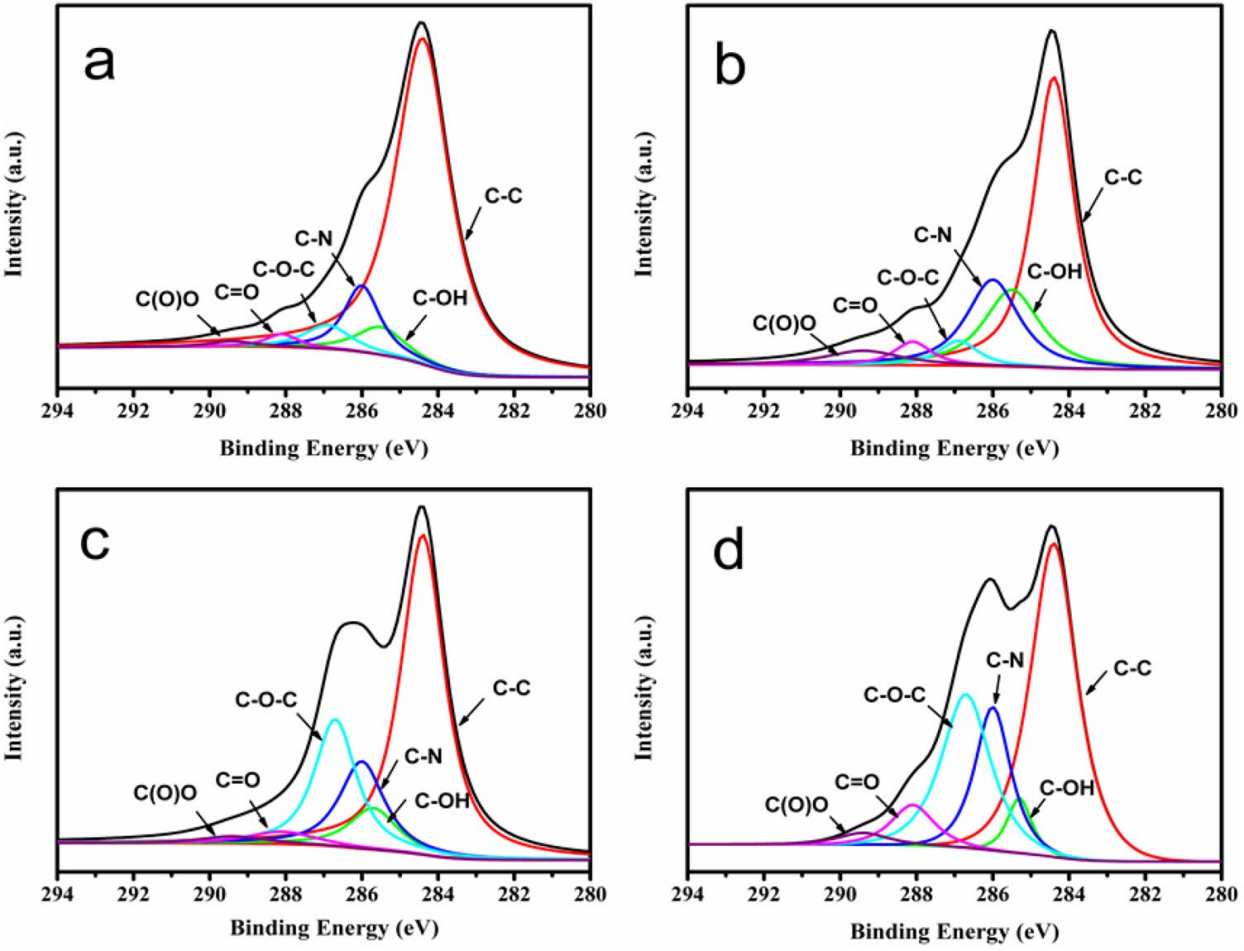
XPS spectra of (**a**) P-GCP-1, (**b**) P-GCP-2, (**c**) P-GCP-3, and (**d**) P-GCP-4 ternary artificial nacre nanocomposites.

**Figure 8:**
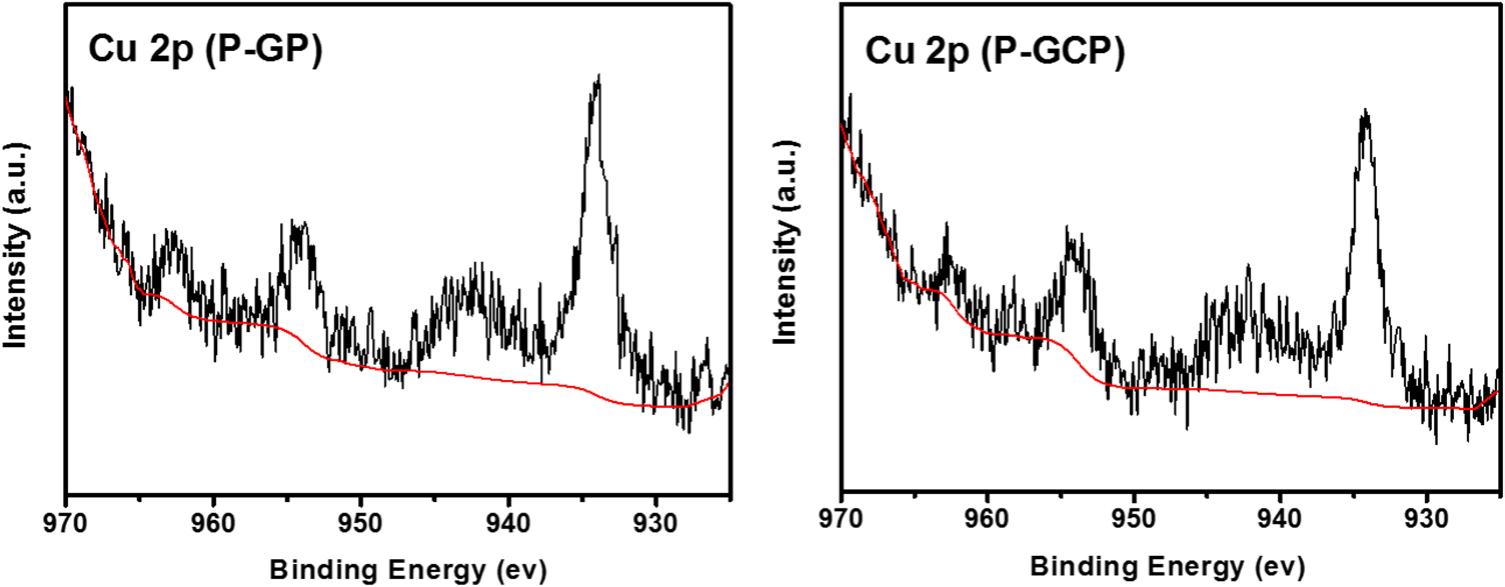
XPS spectrum of Cu 2p in P-GP and P-GCP nanocomposites.

The XPS analysis results of the P-GP binary artificial nacre nanocomposites and P-GCP ternary artificial nacre nanocomposites are shown in Figs. 6, 7 and 8, respectively. The broad C1s peak of P-GP and P-GCP could be divided into six peaks at 284.4 eV, 285.5 eV, 285.8 eV, 286.9 eV, 288.1 eV, and 289.4 eV, corresponding to C–C, C–OH, C-N, C–O–C, C = O, and C(O)O, respectively^29^. A new peak corresponding to the C-N bond at 285.8 eV demonstrated the covalent cross-linking in the P-GP and P-GCP artificial nacres 29. As shown in Fig. 6, with the increase of DA content in the complex (from P-GP-1 to P-GP-4), the intensity of the C-N bonds increased, indicating that GO and PDA reacted with each other to form stable covalent bonds, further confirming the results of FT-IR analysis (Fig. 4). In Fig. 7, as the CNF content increased (from P-GCP-1 to P-GCP-4), the intensity of the C–O–C bonds increased because a large number of hydroxyl groups were attached to the carbon skeleton of CNF and were able to form hydrogen bonds with GO. Moreover, the intensity of the C = O bonds in the composites constantly increased. Two peaks were found on the Cu 2p XPS spectrum, Cu 2p^3/2^ at 934.1 eV and Cu 2p^1/2^ at 954.5 eV, as showed in Fig. 8^19^. The peak at 954.5 eV can be attributed to the copper nanoparticles, indicating that the material's surface was indeed embedded with dense copper nanoparticles, and the peak at 934.1 eV instead of 932.2 eV represented the formation of chelate architecture between copper ions and polydopamine in the composites, which verified that copper plays key roles in enhancing the strength and improving the conductivity in artificial nacre^30,31^.

The elemental contents of the P-GP binary and P-GCP ternary artificial nacres are listed in Table 2, indicating that P-GP and P-GCP contain four elements: carbon, oxygen, nitrogen, and copper. In the P-GP nanocomposites, as the content of PDA increased, the content of carbon decreased. When the amount of PDA added was 5 wt.%, the carbon content of P-GP-1 was 75.87%. When the amount of PDA added was increased to 25 wt.%, the carbon content of P-GP-4 decreased to 69.45%. The nitrogen element mainly comes from GO/PDA film and PDA coating, as the amount of PDA added increased, the nitrogen content of P-GP did not show a rising trend, which was owing to the uneven coating of PDA during the process of preparation. In the P-GCP artificial nacre, as the amount of CNF added increased, the C1 carbon content decreased. Nitrogen also has a downward trend. The C1 carbon content of P-GCP-1 was 69.76%, while that of P-GCP-4 was 49.58%. The nitrogen content of P-GCP-4 is not lower than that of P-GCP-3 It is affected by the uniformity of the coating. In addition, the nanocomposites also contained a small amount of copper, indicating that copper nanoparticles had been embedded on the surface of the material, confirming the results of EDS analysis.

**Table 2:** Elemental contents of P-GP binary artificial nacre and P-GCP ternary artificial nacre.

Sample	Element (PP At. %)			
	C 1s	O 1s	N 1s	Cu 2p
P-GP-1	75.87	18.78	4.46	0.88
P-GP-2	67.98	22.61	8.87	0.54
P-GP-3	67.51	21.18	10.79	0.53
P-GP-4	69.45	24.82	5.53	0.19
P-GCP-1	69.76	20.71	9.06	0.47
P-GCP-2	64.36	29.32	6.02	0.30
P-GCP-3	56.44	37.67	2.58	3.31
P-GCP-4	49.58	44.26	3.42	2.74

**Figure 9:**
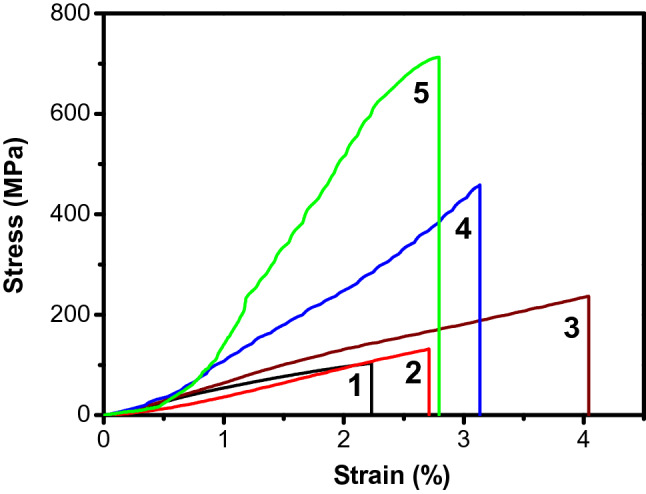
Tensile stress–strain curves of GO (Curve 1), rGO (Curve 2), rGO/PDA (Curve 3), P-GP (Curve 4), and P-GCP (Curve 5).

**Figure 10:**
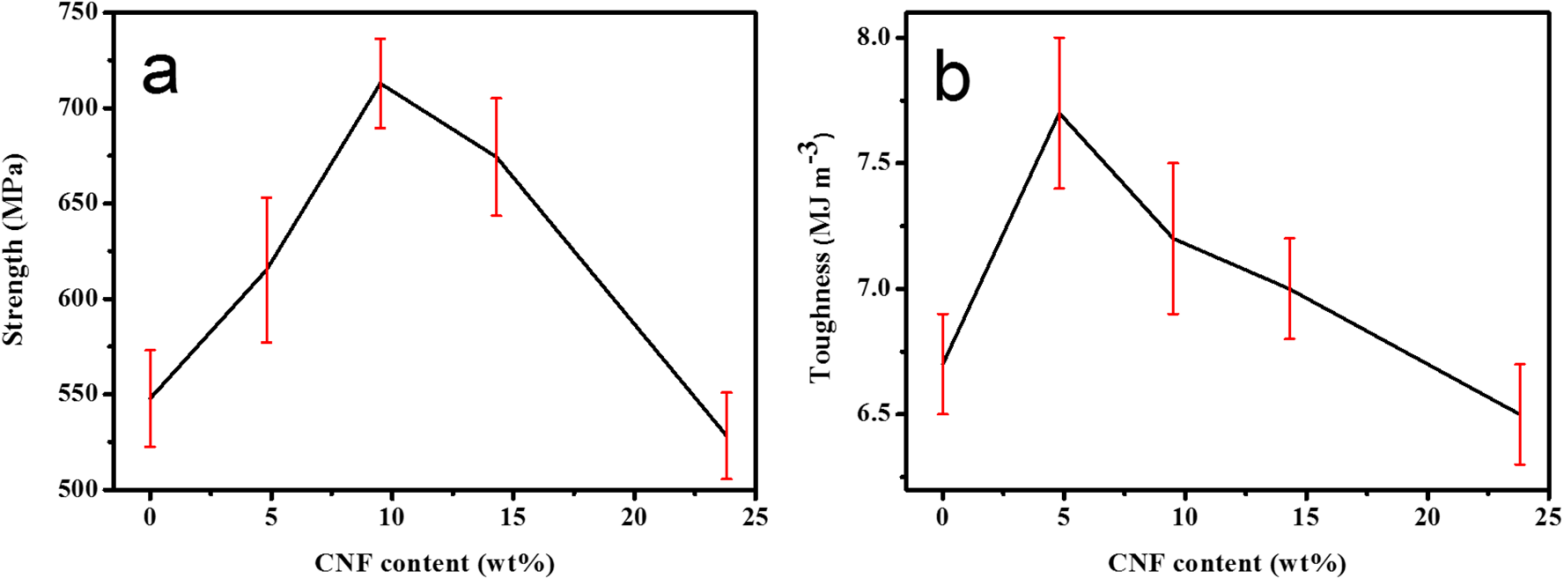
(**a**) Strength and (**b**) toughness of P-GCP artificial nacre with different CNF contents.

The typical tensile stress–strain curves of graphene oxide and related composites are presented in Fig. 9. (The detailed mechanical properties are listed in Table 3.) The tensile strength (106.2 ± 15.3 MPa) and toughness (1.3 ± 0.4 MJ/m^3^) of pure GO film (Curve 1 in Fig. 9) were consistent with the values reported previously^30^. After chemical reduction in HI solution, the reduced graphene oxide film is obtained, and its tensile strength and toughness are slightly improved, reaching 132 ± 15.1 MPa and 2.3 ± 0.3 MJ MJ/m3 This is because graphene oxide introduces a large number of oxygen-containing groups during the preparation process, and these functional groups destroy the excellent mechanical and electrical properties of the material. After chemical treatment, most of the groups are reduced, structural defects are partially repaired, and the mechanical properties of the material are improved. As a type of long-chain polymer, polydopamine can provide high toughness to the material, and the tensile strain of rGO/PDA material is increased to 4.1%. Inspired by the composition of mussel adhesion protein, the surface of GO/PDA and GO/CNF/PDA materials were coated with polydopamine to obtain P-GP and P-GCP artificial nacre materials, with stresses of 459.5 MPa and 712.9 MPa, points increased by 22.0% and 35.2%. But compared to r GO/PDA, the tensile strain of P-GP material is reduced. This is because the unreduced copper ions on the surface react with polydopamine to form a polydopamine-copper ion chelating structure. This special ionic bond can harden the polymer matrix, promote load transfer and increase the hardness of the material. Nanocellulose, a member of one-dimensional rod-like nanomaterials, is often used as a filler for polymer matrix33. With increase in CNF content, the mechanical properties of P-GCP continued to improve (Fig. 10). When the amount of CNF added was 9.5 wt.%, the tensile strength and toughness of artificial nacre reached 712.9 ± 23.4 MPa and 7.2 ± 0.3 MJ/m^3^, which were about 30% and 8%, respectively, higher than that of P-GP. At this time, the synergistic effect between GO and CNF was maximized. As the amount of CNF added continued to increase, the tensile strength and toughness of artificial nacre gradually decreased owing to the effect of excessive CNF on the synergistic effect of artificial nacre. This is because the content of nanocellulose between the sheets is saturated at this time, and excess nanocellulose will accumulate on the surface of the material and affect the load transfer.

**Table 3:** Thicknesses and mechanical properties of P-GCP ternary artificial nacre.

Sample	Thickness (mm)	Strength (MPa)	Modulus (GPa)	Toughness (MJ·m^−3^)
P-GCP-1	0.175	615.1 ± 37.9	7.2 ± 0.6	7.7 ± 0.3
P-GCP-2	0.170	712.9 ± 23.4	8.4 ± 2.1	7.2 ± 0.3
P-GCP-3	0.162	674.3 ± 30.8	9.9 ± 2.3	7.0 ± 0.2
P-GCP-4	0.151	528.2 ± 22.7	7.7 ± 2.5	6.5 ± 0.2

**Figure 11:**
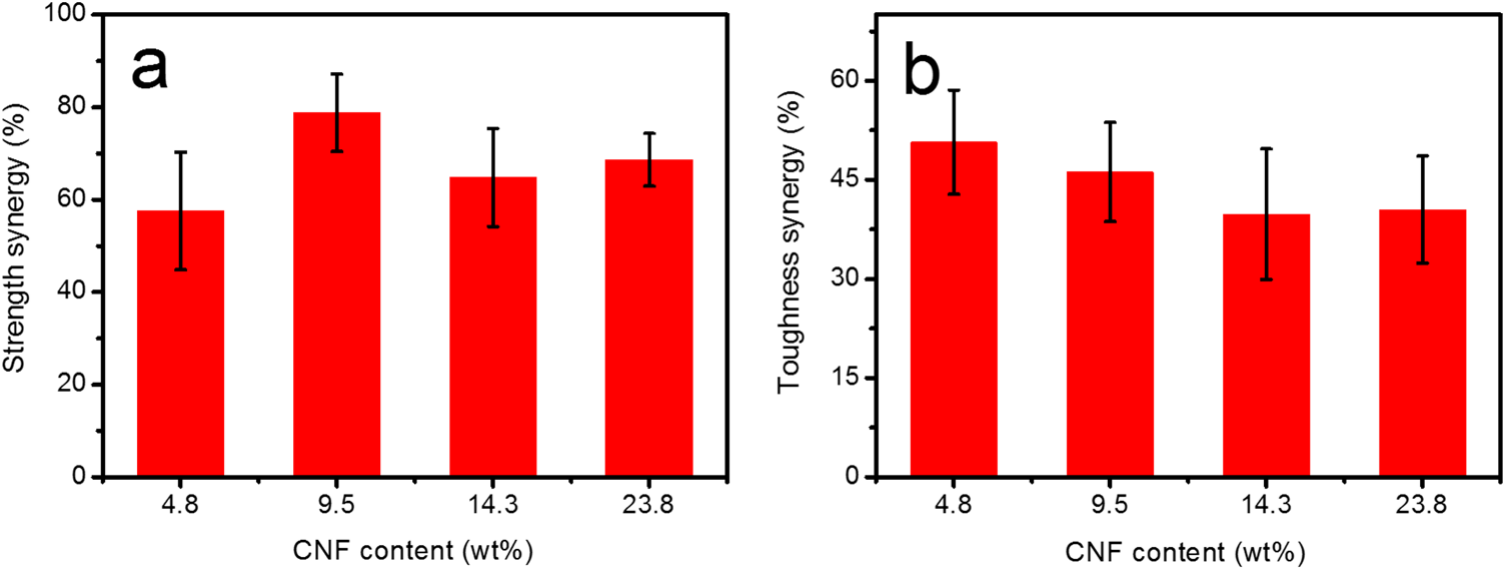
Synergy percentage of increases with CNF contents in P-GCP ternary artificial nacre nanocomposites: (**a**) strength and (**b**) toughness synergy percentage.

The synergy parameter often used to quantitatively evaluate the synergistic interface interactions from covalent and hydrogen bonding, and the synergistic building blocks of GO and CNF as follows

$$S = \frac{{2{M_{{\text{P}} - {\text{GCP}}}} - \left( {{M_{P - GC}} + {M_{P - GP}}} \right)}}{{{M_{P - GC}} + {M_{P - GP}}}} \times 100\%$$

where S is the synergy parameter and MP-GCP, MP-GC, and MP-GP represent the mechanical properties of P-GCP, P-GC, and P-GP nanocomposites, respectively. The mechanical properties of P-GC and P-GP are shown in Table 4. When the amount of CNF added was 4.8 wt.%, the synergistic effect of the artificial nacre was relatively low, the synergistic parameters of strength was 57.6% (Fig. 11). The maximum strength synergy percentage could be achieved with optimal CNF content of 9.5 wt.%. At this time, the synergistic parameter of strength was increased to 78.8% (Table 5), indicating that synergistic effects could be optimized by adjusting the ratios of GO and CNF^18^. Furthermore, this research also confirmed that the synergy percentage could be additionally improved via constructing hydrogen and covalent bonding together, which provided a new inspiration for the preparation of high-strength artificial nacre (Table 6).

**Table 4:** Mechanical properties of P-GP ternary artificial nacre and P-GC ternary artificial nacre.

Sample	Strength (MPa)	Toughness (MJ m^−3^)
P-GP-1	459.5 ± 30.3	5.9 ± 0.4
P-GP-2	413.4 ± 20.6	5.5 ± 0.2
P-GP-3	379.2 ± 22.3	4.8 ± 0.3
P-GP-4	325.1 ± 17.3	4.2 ± 0.3
P-GC-1	321.1 ± 31.8	4.2 ± 0.2
P-GC-2	384.1 ± 21.3	4.3 ± 0.3
P-GC-3	379.2 ± 22.3	5.2 ± 0.2
P-GC-4	301.5 ± 19.6	5.1 ± 0.3

**Table 5:** The synergy percentage of strength and toughness of P-GCP artificial nacre with different contents of CNF.

Sample	Synergy percentage (%)	
	Strength	Toughness
P-GCP-1	57.6	50.7
P-GCP-2	78.8	46.2
P-GCP-3	64.8	39.8
P-GCP-4	68.6	40.5

**Table 6:** Mechanical properties of natural nacre, P-GCP-2 ternary artificial nacre, and other GO-based layered materials.

Layered materials	Strength (MPa)	Toughness (MJ/m^3^)	Reference
Nacre	135.0	1.8	^32^
GO-Mg^2+^	80.6	0.1	^16^
GO-GA	101.0	0.3	^33^
GO-Ca^2+^	125.8	0.3	^16^
rGO-PCDO	129.6	3.9	^34^
GO-PMMA	148.3	2.4	^35^
GO-Borate	185.0	0.1	^36^
rGO-PVA	188.9	2.5	^37^
rGO-PDA	205.0	4.0	^26^
GO-PEI	209.9	0.2	^38^
rGO-MoS_2_-TPU	235.0	6.9	^25^
rGO-HPC-Cu	274.3	6.7	^39^
rGO-Cu	284.0	2.2	^40^
rGO-SL	300.0	2.6	^41^
rGO-PAA	309.6	8.9	^42^
rGO-MMT-PVA	356.0	7.5	^43^
rGO-DWNT-PVA	375.8	11.3	^44^
rGO-PAPB	382.0	0.2	^45^
rGO-PDA-Ni	417.2	19.5	^29^
rGO-PCDO-Zn	439.1	7.6	^46^
GO-CNC	490.0	3.9	^47^
rGO-CNC	655.0	2.0	^47^
GO-CS-Cu	868.6	14.0	^30^
SBG	944.5	20.6	^48^
P-GCP-2	712.9	7.2	This work

**Figure 12:**
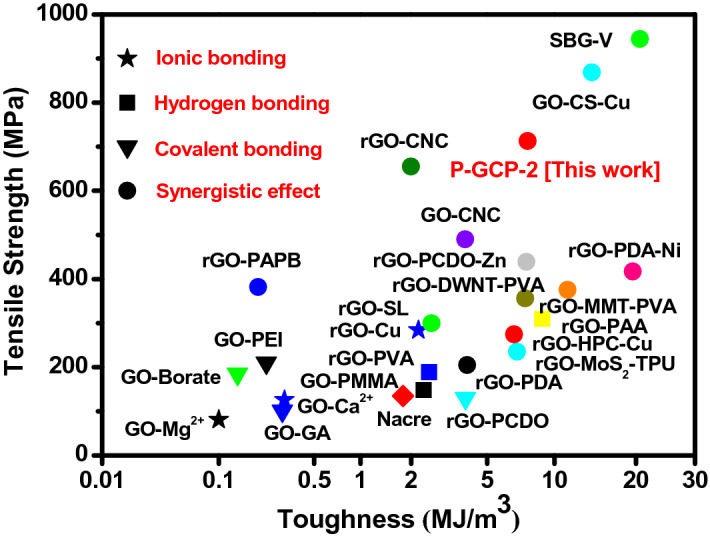
Comparison of tensile strength and toughness of P-GCP-2 ternary artificial nacre with those of other layered materials.

Compared with natural nacre and other graphene/graphene oxide-based nanocomposites, P-GCP-2 revealed an overwhelming superiority is shown in Fig. 12, and detailed parameters are listed in Table 5. GO nanosheets are linked with different molecules in synergistic effect, such as rGO-silk fibroin (SL)^41^ and rGO-montmorillonite (MMT)-PVA^43^. In general, the synergistic effect of artificial nacre results in better mechanical properties than those obtained from single interface interaction including hydrogen bonding with GO-poly(methyl methacrylate) (PMMA)^37^ and rGO-PAA^42^, ionic bonding with GO-Mg^2+^^16^ and rGO-Cu^40^, covalent bonding with GO-glutaraldehyde (GA)^33^, and GO-polyetherimide (PEI)^38^. Compared with natural nacre, P-GCP artificial nacre has excellent mechanical properties, and its tensile strength and toughness reach 712.9 MPa and 7.2 MJ/m^3^, which are 5.3 and 4.3 times of natural nacre, respectively. The rGO-based composites reinforced with copper (rGO-Cu) have high mechanical strength and toughness of 284 MPa and 2.2 MJ/m^3^, respectively. However, these values were only 39% and 31% of those of P-GCP artificial nacre. The toughness values of GO-PCDO with covalent bonding and GO-PDA with synergistic effect were 3.9 MJ/m^3^ and 4.0 MJ/m^3^, respectively, which were both slightly lower than that of P-GCP artificial nacre.

Recently, Cheng et al. presented bioinspired graphene-based nanocomposites, rGO-CS-Cu, which improved the record of tensile strength to 868.6 MPa^30^. Sequentially bridged graphene (SBG) composites reported by Wan et al., which had an realized tensile strength of 945 MPa^48^. Although the tensile strength of the above composites is higher than that of P-GCP, the role of novel micro/nano architecture should not be ignored. The rGO/PDA composites reported initially had a tensile strength of 205 MPa^26^. After building the micro/nano architecture, the tensile strength of the related nanocomposites increased by 2.5 times. Therefore, if applied to rGO-CS-Cu or SBG composite materials, it is believed that the record of mechanical properties would be further improved.

**Figure 13:**
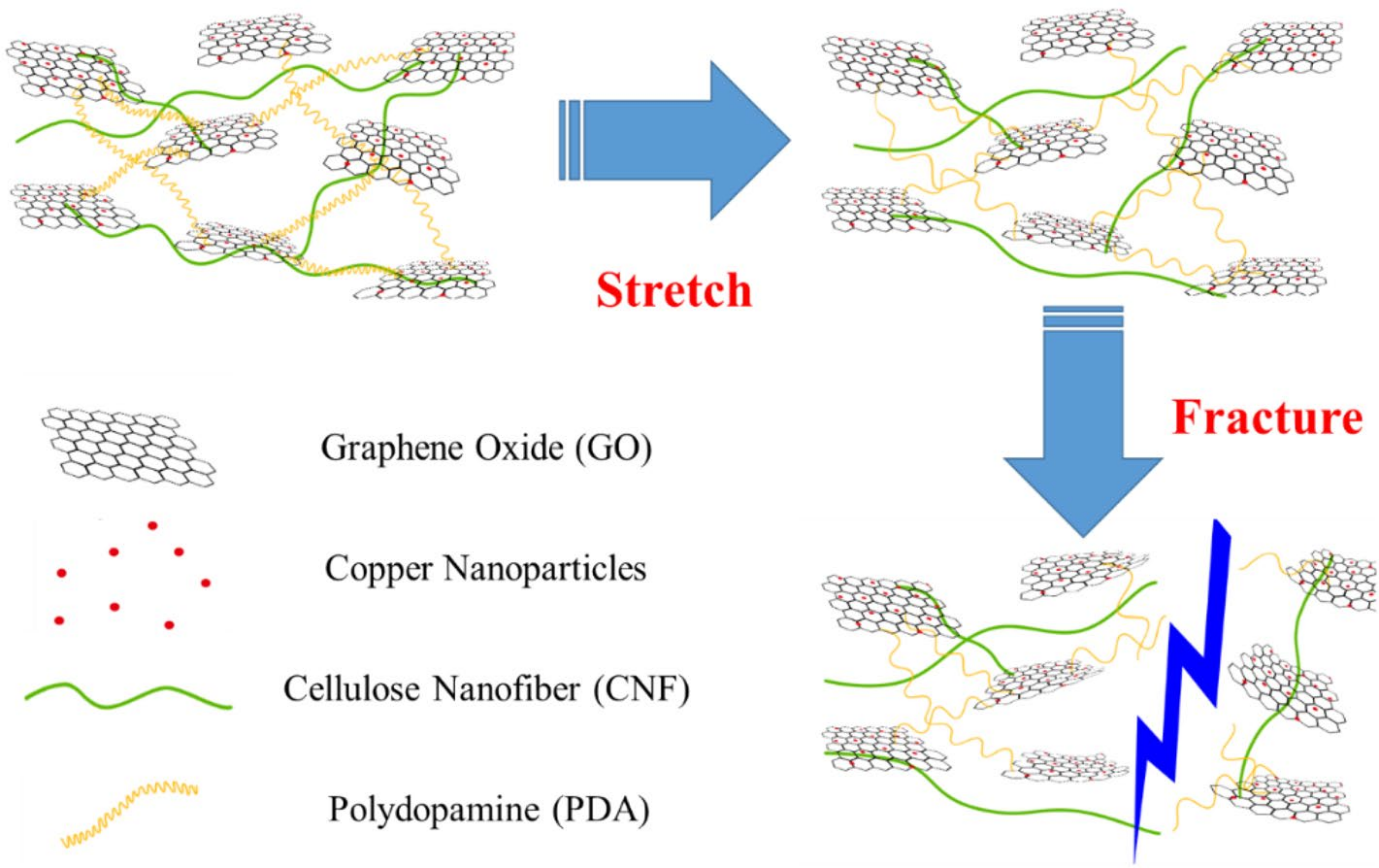
Proposed synergistic mechanism of ternary P-GCP artificial nacre.

**Figure 14:**
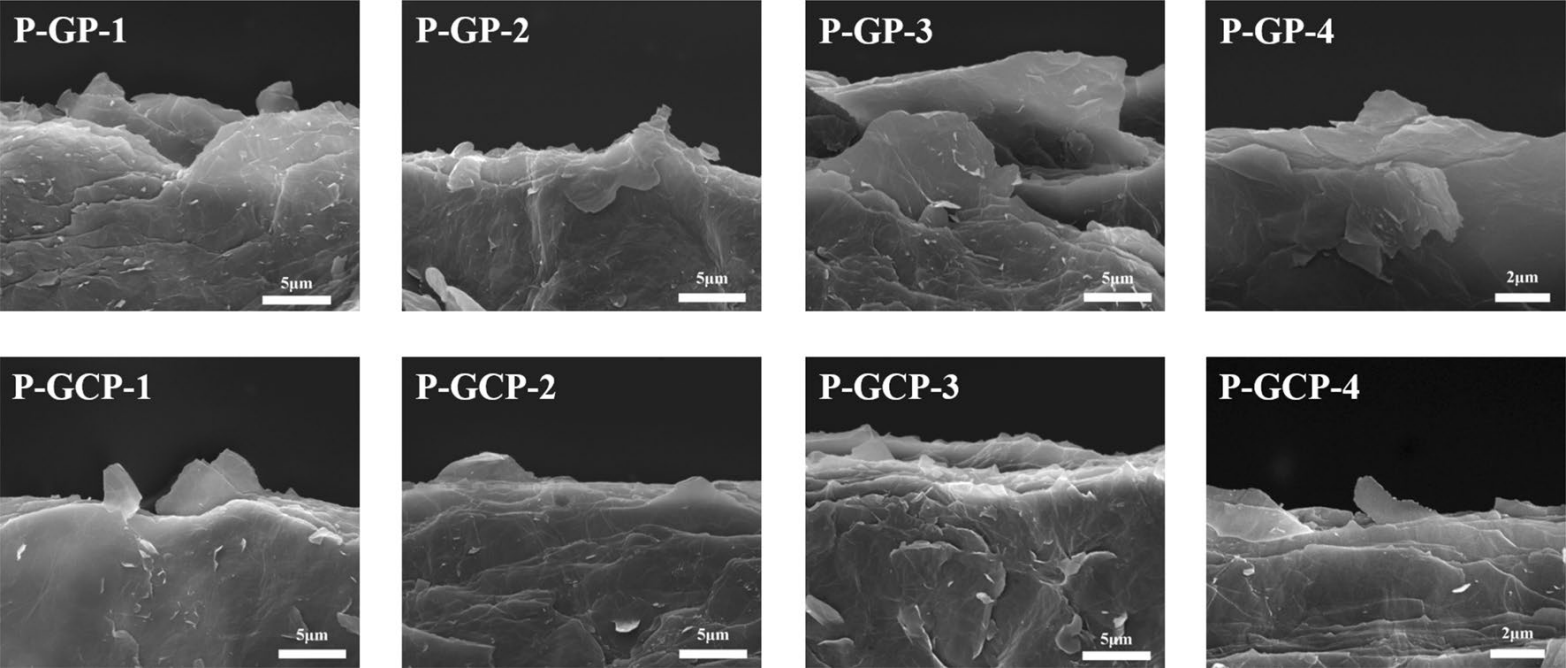
Fracture surface morphologies of P-GP and P-GCP artificial nacres.

A crack extension model was proposed to further understand the tensile fracture mechanism of P-GCP artificial nacre, as shown in Fig. 13. In the initial stage, the P-GCP artificial nacre contained various bonds, such as weak hydrogen bonds between the GO sheets, hydrogen bonds between GO and CNF, covalent bonds between GO and PDA, and poly(dopamine)-copper ion chelate architecture. At this time, CNF and PDA were in curled and coiled states. After being subjected to loading stress, the short and weak bonds between the nanosheets were first affected, such as hydrogen bonds and ionic bonds, and they were broken after exceeding the tolerance range. In the second stage, the stress was concentrated on the poly(dopamine)-copper ion chelate architecture. Similarly, when the stress reached a certain level, the chelate architecture was destroyed and the adjacent GO nanosheets were relatively slipped. As tensile stress increased, all the short and weak bonds were exhausted, and the relative displacement between the nanosheets was further increased. When the strain reached a certain level, the long chain of polydopamine and the molecular chain of nanocellulose were straightened and the strain was no longer increased. During the tensile fracture stage, the covalent bonds between GO and PDA was destroyed, and cracks appeared on the surface of the composites until fractured. At various stages of fracture failure, a large amount of energy was absorbed or consumed, which increased the tensile strength and toughness of the composites. On the fracture surface of the artificial nacre, it was found that the GO nanosheets were bent, as shown in Fig. 14. Therefore, the hydrogen bonds, ionic bonds, and covalent bonds were sequentially broken, which synergistically enhanced the mechanical properties such as tensile strength and toughness of the P-GCP ternary artificial nacre^24^.

**Figure 15:**
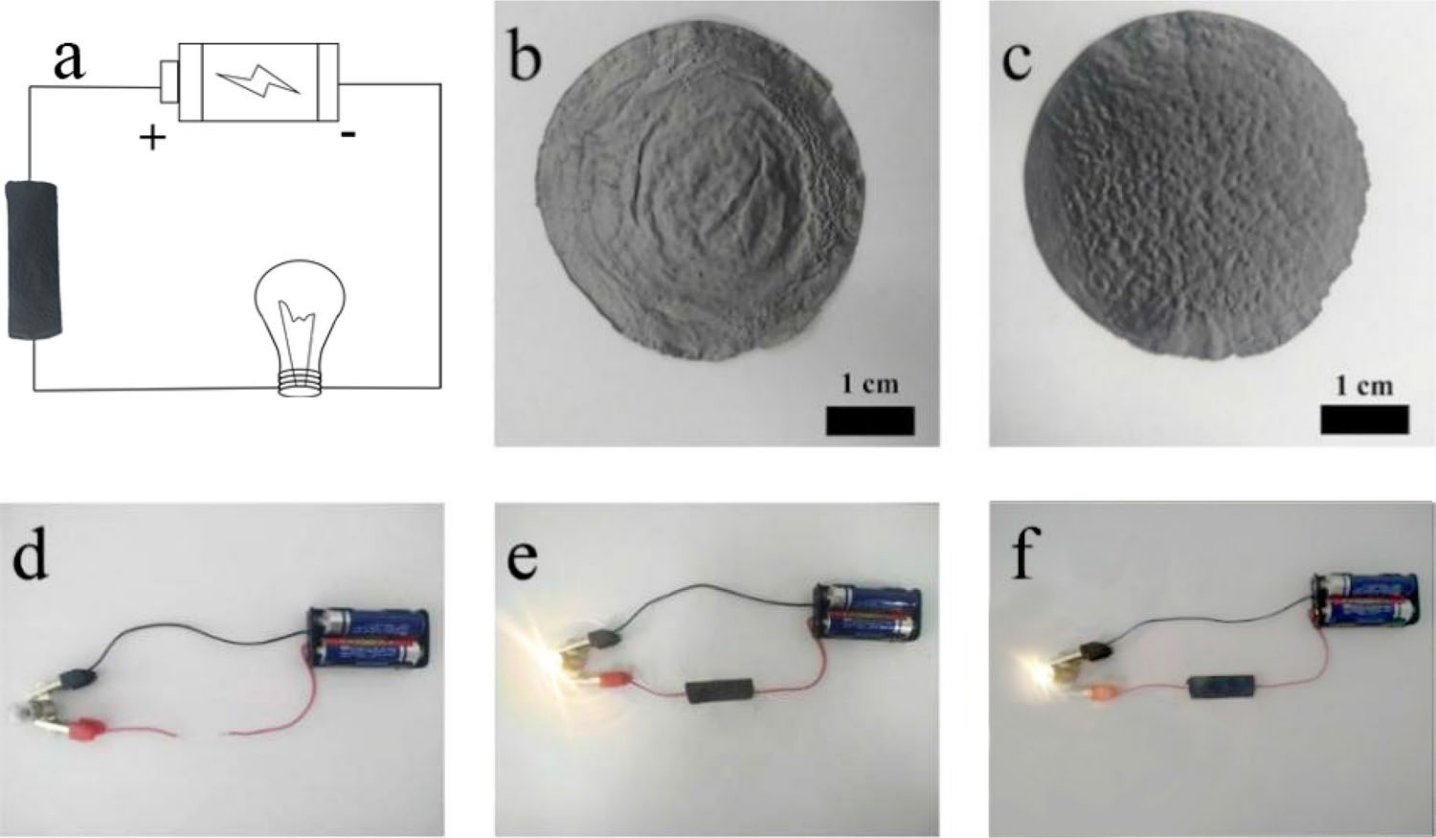
(**a**) Schematic of the circuit. (**b**) rGO film. (**c**) P-GCP ternary artificial nacre. (**d**) The digital photo of circuit. (**e**) The rGO film, (**f**) P-GCP ternary artificial nacre as a part of conductive media connected with power supply and loaded with a bulb.

In addition to excellent mechanical strength and toughness, the electrical properties of artificial nacre were equally impressive (Table 7). rGO obtained after reduction with chemical reagents such as hydroiodic acid exhibited excellent electrical conductivity, 224.9 S/cm, which was consistent with that previously reported^30^. From the content of copper ions in Table 2 and the ratio of PDA, GO and CNF during preparation, the CNF content in the composite material can be obtained as shown in Table 7 below. As a kind of reinforcement material, CNF has almost no conductivity, and excess CNF will accumulate on the surface of the rGO sheets, thus affecting the electrical properties of the composites. In previous work, we reported ternary artificial nacre, rGO/CNF/PDA^49^. However, the electrical conductivity of composites was only 56.2 S/cm. Aiming at improving electrical performance, P-GCP was prepared. When PDA coating and copper nanoparticles are introduced into the composites, the effect of insulator on conductivity can be reduced due to the presence of copper nanoparticles, when the content of nanocellulose was 4.8%, the electrical conductivity reached 207.6 S/cm, which was equivalent to that of rGO. Compared with the previous research results, it can be found that the addition of copper does increase the conductivity of the composite material. In the samples from P-GCP-1 to P-GCP-4, as the content of rGO gradually decreases and CNF increases, the conductivity of the composite material gradually decreases. But under the condition that the content of CNF increases in the same proportion, from Tables 2 and 6, the conductivity difference between P-GCP-1 and P-GCP-2 is significantly larger than that between P-GCP-2 and P-GCP-3. This is because there is less copper in P-GCP-2. Building a circuit, using a yellow bulb connected to the power supply, and using nanocomposites as a conducting wire, the electrical conductivity of the P-GCP ternary artificial nacre was investigated (Fig. 15). The test results indicated that the P-GCP artificial nacre had the same excellent electrical conductivity as the rGO film and had significant potential for application in the fields of aerospace engineering, artificial muscles, and tissue engineering.

**Table 7:** Electrical properties of rGO film and P-GCP ternary artificial nacre nanocomposites.

Sample	Input CNF content [wt%]	Electrical conductivity (S cm^−1^)
rGO	–	224.9 ± 8.6
P-GCP-1	4.8	207.6 ± 13.1
P-GCP-2	9.5	194.4 ± 15.2
P-GCP-3	14.3	186.7 ± 10.3
P-GCP-4	23.8	169.6 ± 18.9

## Conclusion

Inspired by the combination of various creatures, we proposed a novel graphene-based micro/nano architecture, and prepared P-GCP by a vacuum-assisted filtration self-assembly process. Based on the "brick–mortar" structure of natural nacre, nanocomposites with hierarchical structure were designed. Inspired by the hardening mechanism of Glycera’s jaw and the adhesive proteins in mussels, the mechanical and electrical properties of materials were additionally improved. During the tensile fracture process, the synergistic effects between hydrogen bonding, ionic bonding, covalent bonding, and chelate architecture increased the strength to 712.9 MPa. The connection of P-GCP in the circuit reflected the high conductivity (207.6 S/cm), which demonstrated the practical application prospects in aerospace, supercapacitors, biomaterials, artificial bones, and tissue engineering.
